# “Reluctant to Assess Pain”: A Qualitative Study of Health Care Professionals’ Beliefs About the Role of Pain in Juvenile Idiopathic Arthritis

**DOI:** 10.1002/acr.23827

**Published:** 2019-12-27

**Authors:** Rebecca Rachael Lee, Amir Rashid, Wendy Thomson, Lis Cordingley

**Affiliations:** ^1^ Manchester University Hospitals NHS Foundation Trust Manchester Academic Health Science Centre The University of Manchester Manchester UK; ^2^ Manchester Academic Health Science Centre The University of Manchester Manchester UK

## Abstract

**Objective:**

Reducing pain is one of the main health priorities for children and young people with juvenile idiopathic arthritis (JIA); however, some studies indicate that pain is not routinely assessed in this patient group. The aim of this study was to explore health care professionals’ (HCPs) beliefs about the role of pain and the prioritization of its assessment in children and young people with JIA.

**Methods:**

Semi‐structured interviews were conducted with HCPs who manage children and young people with JIA in the UK (including consultant and trainee pediatric rheumatologists, nurses, physical therapists, and occupational therapists). Data were analyzed qualitatively following a framework analysis approach.

**Results:**

Twenty‐one HCPs participated. Analyses of the data identified 6 themes, including lack of training and low confidence in pain assessment, reluctance to engage in pain discussions, low prioritization of pain assessment, specific beliefs about the nature of pain in JIA, treatment of pain in JIA, and undervaluing pain reports. Assessment of pain symptoms was regarded as a low priority and some HCPs actively avoided conversations about pain.

**Conclusion:**

These findings indicate that the assessment of pain in children and young people with JIA may be limited by knowledge, skills, and attitudinal factors. HCPs’ accounts of their beliefs about pain in JIA and their low prioritization of pain in clinical practice suggest that a shift in perceptions about pain management may be helpful for professionals managing children and young people with this condition.

## Introduction

Juvenile idiopathic arthritis (JIA) is a chronic inflammatory arthritis diagnosed in children and young people <16 years of age 1. Chronic pain is one of the most common features of this long‐term condition 2, 3, 4, 5, 6 and many studies have demonstrated that pain has a high daily prevalence in JIA 7, 8. Managing JIA‐related pain can be both a challenge and a burden for children and young people, as it can interfere with multiple aspects of everyday life 9 including physical, social, and academic activities 10.

**Significance & Innovations**

- Health care professionals reported gaps in pain‐specific knowledge and skills to assess and manage pain in children with juvenile idiopathic arthritis (JIA).
- Beliefs about the occurrence of pain in the context of JIA contributed to a reluctance to prioritize pain assessment by some rheumatologists, nurses, physical therapists and occupational therapists.
- Therapists were more likely than rheumatologists to express concerns about an “over‐medicalized” approach to treatment of JIA.
- A paradigm shift in approaches to pain assessment and communication by professionals managing those with JIA may be useful to improve both pain management and pain communication with patients and families.

In a thematic synthesis of the experiences of children and young people living with JIA, Tong et al 11 found that the invisible nature of pain was described as the “worst thing” about living with the condition. In another study, patients viewed opportunities to describe the course of pain in JIA as high priority, whereas health care professionals’ (HCPs’) views did not correspond 12. Some authors suggest that HCPs regularly overlook the assessment of pain in children with long‐term conditions 13, 14, however it is not clear why this might be the case. This situation is problematic because the presence of pain in children with JIA is not fully explained by disease activity alone 2, 15. Chronic pain continues to be a burden even throughout periods when underlying disease processes are controlled with medication and disease activity is low 5, 7, 16, 17, 18, 19. Furthermore, HCPs have been poor at predicting levels of pain in children with JIA, sometimes providing overestimated (i.e., worse) ratings than children themselves 20 and sometimes underestimations 21. The nonlinear relationship between pain and disease activity, taken together with HCPs inability to accurately estimate pain levels, suggests that a separate assessment of pain is necessary and should include self‐report of pain symptoms by children.

Pain assessment provides the basis upon which to develop, refine, and evaluate pain management strategies and is necessary to achieve improvements in pain symptoms 22. A full pain assessment requires attention to intensity, frequency, location, and interference, information which should inform JIA care decisions 10. Despite many authors advocating thorough pain assessment in JIA, there is little published literature investigating HCPs attention to pain in this long‐term condition. The aim of the current research study was to explore HCPs’ beliefs about the role of pain and the prioritization of its assessment in professionals involved in the management of children and young people with JIA throughout the UK.

## Materials and Methods

The research that was carried out was in compliance with the Helsinki Declaration. Ethical approval was granted by the authors’ institutional research ethics committee (ref: 15454). HCPs were recruited via a study advertisement circulated by The British Society for Paediatric and Adolescent Rheumatology, a professional membership organization 23. HCPs were eligible for the study if they worked in the UK National Health Service as pediatric rheumatologists (either consultant or trainee), pediatricians, nurse specialists, physical therapists, or occupational therapists managing children and young people with JIA.

A data‐driven inductive approach was chosen to guide data collection and analysis as there was no predefined theory about pain assessment and communication in UK pediatric rheumatology settings. Interviews were conducted either face‐to‐face or over the telephone and all were digitally audio recorded. Interviews were semistructured and followed the format and questions outlined in the interview topic guide (Table 1). The first draft of the interview topic guide was developed among the authors, mainly using observations of clinical consultations between pediatric rheumatologists and patients with JIA. During the observations of HCPs in the clinic of 1 author (RRL), notes were made about issues discussed with patients, explanations of disease or pain, attention given to assessment, and specific advice given about treatment. These observations were mapped onto existing rheumatology and pain‐specific literature (including research and clinical guidelines) about pain assessment and management issues in each field. Contrasts in the pain assessment approaches in the fields of pain and rheumatology were used to highlight specific problems. Issues and problems raised through observations and research articles were then developed into questions by the study team.

**Table 1:** Final topic guide used in semistructured interviews^a^

Questions	Probes and prompts
What is your experience of working with individuals with JIA?	Current role and past roles? Frequency of contact with patients with JIA? Training in assessment and management of JIA/pain?
How/what do you consider the role of pain to be in JIA?	What is the importance of pain for CYP with JIA? What is the relationship of pain with disease activity?
What do you think are the main influences/causes upon the amount/severity of pain an individual with JIA suffers from?	What are the biomedical influences? What are the biopsychosocial influences?
Do you believe that significant attention to pain is given in clinical consultations between health care professionals and CYP with JIA?	Could you tell me about some of your own experiences of addressing pain in these patients? Can you tell me about any possible reasons or scenarios in which pain is/is not significant to consider?
Do you routinely assess pain as part of your clinical appointments with patients?	What do you tend to focus on? What do you tend to not spend much time covering?
How do you communicate with patients with JIA about pain symptoms?	How is the topic of pain approached? Are there any particular barriers to talking about pain? What helps you and patients to communicate about pain? Do you use any particular scales? Do you think the reporter of pain is important? Are there any facets of pain information which you find to be more important than others (e.g., information about intensity, location, frequency)?
To what extent do you think information about pain is used to guide management/treatment decisions in JIA?	How does pain information affect your treatment/management decisions? What would you adapt in your treatment/management plan of patients based on pain reports?
What advice do you give patients about pain management?	What advice do you give regarding the impact of pain? What advice do you give if patients complain of their joints being painful?
To what extent do you think pain assessment is conducted in current practice?	What issues do you envisage with aiming to make pain assessment better in practice?
Do you use any particular guidelines or policy documents for measuring progress in JIA?	What guidelines are you aware of? What is your opinion on the appropriateness/inappropriateness of outcomes measured?
How do you think we can make pain assessment better for CYP with JIA?	Do you think any particular tools would be helpful? Or any particular resources helpful? Do you think particular teams of professionals are helpful to have involved in assessment and pain management?
Do you think pain assessment is conducted similarly between all groups of health care professionals?	Do you think different professional teams are better placed to assess pain? Do you think there is a difference in pain assessment approaches between people working in the same profession?

a JIA = juvenile idiopathic arthritis; CYP = children and young people.

The interview topic guide was refined after piloting with a trainee pediatric rheumatologist. Audio recordings of interviews were transcribed verbatim. All audio recorded interviews were uploaded to and analyzed in NVivo 10 (QSR International).

The framework analysis method 24 was adopted. The 5 stages of conducting framework analysis include familiarization, identification of a thematic framework, indexing, charting, and mapping/interpretation. One author (RRL), an experienced qualitative analyst with a research background in Health Psychology, served as the main analyst and coded all of the data in NVivo using audio recordings and interview transcripts simultaneously. Data collection was paused after the first 6 interviews as a familiarization exercise for the main data analyst, to ensure the data being captured was in line with the aims of the study and so that the interview guide could be modified if the data collected was not appropriate. Two other members of the research team (AR and LC) reviewed audio recordings, transcripts, initial themes, and interpretations produced from the main data analyst at this stage of the analysis. Disagreements (e.g., the meaning of participant quotations and how they mapped onto themes in the initial index) were addressed through group discussions until clarity and consensus were obtained. The interview guide was not modified following this because questions were considered to be appropriate and data capture was relevant to the aims of the study (as agreed upon by all authors).

During this data collection pause, an initial index of themes was developed in line with the framework analysis approach 24 by the main data analyst. The initial index portrayed a priori issues (reflecting the research aims and questions posed in the interview guide) as well as new issues raised by participants and recurring patterns of views in data. The initial index included categories of early emerging themes and was used to examine, sort, and guide the interpretation of interview data. The initial index was refined and developed further as the remaining interview data were collected and interpreted. New themes were added to the initial index, and the interpretations of initial themes were adapted based upon new evidence. The process of refining and developing the initial index was conducted with 2 other members of the research team (AR and LC) until a thorough index that could be applied to participant data was created (see Supplementary Table 1, available on the *Arthritis Care & Research* web site at http://onlinelibrary.wiley.com/doi/10.1002/acr.23827/abstract).

Using the index, data were arranged hierarchically into themes and subthemes by the main analyst (RRL). A matrix output was generated once all interview transcripts had been organized into these themes/nodes 25. Comparisons were made between participants’ accounts to look for meaning and connections. These connections were then organized, grouped, and mapped using the index and were then interpreted into overarching themes and narratives. Two other members of the research team (AR and LC) reviewed the organized data, emerging themes, and narratives for clarity and meaningfulness. Themes were reorganized and reinterpreted as part of some discussions between the research team. When there were any apparent gaps in the themes identified, interview transcripts were revisited for additional coding to support and/or refute the interpretations generated from the data.

After 15 interviews, the materialization of new information from participants plateaued. Six planned interviews were carried out to ensure that data saturation had occurred. Despite ongoing interest from 5 professionals, no further interviews were planned.

## Results

#### Participant demographics

Twenty‐one HCPs participated, working in 12 different pediatric rheumatology departments across England and the Republic of Ireland (Table 2). Interview times ranged from 28 to 65 minutes (mean = 45 minutes). The sample of HCPs included 19 female and 2 male, which is representative of rheumatology departments in the UK (latest estimates indicate 94% of staff are female) 26. Six interviews were conducted face‐to‐face and 15 interviews were completed over the telephone.

**Table 2:** Participant characteristics

Health care profession	Experience, years (range)	Interview type		Sex		Total no. of participants
		Telephone	Face‐to‐face	Female	Male	
Pediatric rheumatologists^a^	1.5–20	6	2	7	1	8
Pediatricians	2	0	1	1	0	1
Nurses	1–5	3	0	3	0	3
Physical therapists	0.5–12	3	3	5	1	6
Occupational therapists	0.25–14	3	0	3	0	3

a Including 2 trainees.

#### Themes and interpretation

Six overarching themes were identified: 1) training, confidence, and competencies in pain assessment; 2) reluctance to engage in pain discussions; 3) low prioritization of pain assessment; 4) beliefs about pain in JIA; 5) treatment of pain; and 6) undervaluing pain reports. Each of these themes will be discussed as a narrative account in subsequent tables.

#### Theme 1: Training, confidence, and competencies in pain assessment (Table 3)

**Table 3:** Themes 1 and 2, subthemes, and associated interview excerpts^a^

Themes and subthemes	Interview excerpts
Theme 1: Training, confidence, and competencies in pain assessment
Subthemes	
There is little/no formal training in how to assess chronic pain. Pain education may occur in medical school but these skills are forgotten about in later training stages.	“No, I haven't had particular pain teaching. We probably did in medical school, but I can't really remember” (Participant 3, pediatrician). “I don't think anyone has very good pain training. I think because it's something that is developing and coming about” (Participant 5, consultant pediatric rheumatologist). “I've been on nothing very specific to JIA and pain assessment… there's nothing out there” (Participant 6, nurse).
Pain assessment knowledge is acquired through observation of other departmental practices.	“It's more just shadowing of what techniques are being used” (Participant 16, occupational therapist).
The lack of pain training leads to low confidence in assessment.	“Some people haven't done any pain training…they feel uncomfortable asking” (Participant 5, consultant pediatric rheumatologist).
Rheumatologists do not consider themselves best placed for pain assessment and/or communication because they do not ask the right questions.	“We as doctors are not necessarily good at exploring pain, we don't ask the right questions. And the other thing is whether or not we as doctors sat in clinic prescribing medicines are the right people to be assessing pain. Have we been trained properly, no, we've not been trained at all” (Participant 9, consultant pediatric rheumatologist).
There is a perception that therapists are best placed for pain assessment because they have more time, are less formal and have more regular contact with patients. Therapists feel that patients with pain are sent to them because rheumatologists do not have the skills to address pain.	“I think allied health professionals ask the same sort of questions as the rheumatologists…perhaps we have more time and it might not feel as formal… we might be seeing them more regularly” (Participant 2, occupational therapist). “And I think that is a lot because they don't feel like they have the skills, so they send them to us” (Participant 13, physical therapist).
Theme 2: Reluctance to engage in pain discussions	
Subthemes	
Evaluation of children's pain is done without asking directly about it. Pain experiences are noticeable through discussions about other aspects of the condition.	“Very seldom do I ask a child about their pain” (Participant 11, physical therapist). “I get a feel for how much pain they've been in. Do I need more information about their pain? I'm not sure I do” (Participant 7, consultant pediatric rheumatologist).
Asking about pain may lead to amplification of pain through exaggerated responses or heightened perceptions.	“Indirectly, I get the information anyway, without saying, are you in pain, which feels like I'm leading them into saying, yes I am” (Participant 2, occupational therapist). “That's what they'll ask, are you in pain? And you know what children are like. Yeah, yeah. They're not really. They're running around, left, right and centre” (Participant 10, nurse).
HCP's fear making the pain worse by drawing attention to it (physically or emotionally).	“Eventually if you're asked enough times, well, yes, maybe I do have pain….nagging them about pain isn't necessarily in their best interest… we educate them about pain and suddenly they all have it” (Participant 5, consultant pediatric rheumatologist).
Pain assessment and discussions can lead to poorer well‐being.	“I don't ask in every consultation because that can be quite demoralising, demoting” (Participant 11, physical therapist).
Managing reported pain is difficult; there are a lack of resources and time.	“Some people don't ask about pain because they don't want to get stuck with having to deal with it” (Participant 5, consultant pediatric rheumatologist). “It's alright me asking about their pain, but then do I have the facility to deal with it?” (Participant 3, pediatrician). “I think the perception of pain is often one whereby people seek to avoid it. I hope it's not because of ignorance…they're just not wanting to open that can of worms. I think it's more related to work based pressures and time” (Participant 20, physical therapist).

a JIA = juvenile idiopathic arthritis; HCP = health care professional.

Participants reported that they had had little or no training on how to assess chronic pain symptoms in children and young people with JIA, and that pain was not an explicit part of rheumatology training. Pain education may have occurred as part of general medical training under the premise that additional knowledge and skills would be picked up in practice by individuals specializing in the field of pediatric rheumatology. Participants indicated that they only learned how to assess and manage pain symptoms in JIA by following current departmental practices. Participants perceived a lack of availability of pain‐specific knowledge or skills training at later stages of their careers.

Participants reported their low levels of confidence in approaching and talking to children and young people about pain experiences because of their lack of pain training. Some felt that other HCP groups were more equipped for this task or perceived other HCPs as having the specific competencies needed for assessing pain in this group. An issue that recurred in several interviews was the idea that therapists, such as occupational or physical therapists, were best placed for assessing pain. Rheumatologists generally discounted themselves as being the best placed for assessing pain because they did not know how to ask the right questions about pain and were not trained to do so. In the accounts provided by therapists, their perceived suitability was linked to the additional time available, less formal consultations and more regular contact with patients, as well as their specific skill sets relating to pain assessment. Therapists believed that referrals that they received to manage patients with pain could sometimes be due to rheumatologists not having the skill set required to address pain without therapy input. Although therapists discussed being best placed to assess pain, it transpired in later themes that there were several other factors that appear to contribute to the lack of pain assessment by allied health professionals.

#### Theme 2: Reluctance to engage in pain discussions (Table 3)

A reluctance to directly elicit information from patients about their pain was found in some of the interviews conducted. Some participants indicated that explicit assessment was not necessary in order to assess pain levels of individuals with JIA. Rather, participants in the current study believed that they could make sense of pain of a child or young person by how they reported on other aspects of their condition, such as joint stiffness. Here, participants felt able to make judgments about pain levels without direct reports from their patients.

Another reason why participants did not ask about pain was because some HCPs feared that there would be undesirable consequences of doing so. There were some concerns that patients may exaggerate or misrepresent their pain experiences simply because they were being asked about pain, or because by putting pain assessment on the agenda patients would feel obliged to report it. Furthermore, participants thought that discussions about pain or a focus on symptoms may lead to children and young people feeling more pain as a consequence of the consultation. Finally, participants discussed how conversations about pain were by their nature “depressing” and could possibly worsen well‐being or lower patient motivation for self‐management.

HCPs rarely asked for pain information from patients directly in clinical consultations, which (as discussed) demonstrates a reluctance to approach and seek information about pain from a HCPs perspective; however, a notable finding in the current study is that even when patients themselves brought up the topic of pain in consultations, some HCPs would actively avoid engaging in conversations. Some participants reported that they would purposefully not become involved in discussions about pain because they were aware that they did not have the resources to address pain management. The difficulties associated with addressing the root cause of pain acted as a deterrent to approaching the subject.

#### Theme 3: Low prioritization of pain assessment (Table 4)

**Table 4:** Themes 3 and 4, subthemes, and associated interview excerpts^a^

Themes and subthemes	Interview excerpts
Theme 3 : Low prioritization of pain assessment
Subthemes	
Low prioritization of pain assessment in clinical consultations	“I don't think we should dwell on pain” (Participant 2, occupational therapist). “I wouldn't ask at every appointment, how has your pain been? I very much try to work away from that” (Participant 4, physical therapist). “So we tend to not to, I suppose, prioritise pain so much” (Participant 8, consultant pediatric rheumatologist). “It's an important factor, but it's not the first on the list” (Participant 6, nurse).
Other priority assessments include measures of function and disease activity	“It's stiffness, lack of function, and lack of movement you're looking for” (Participant 11, physical therapist). “In clinical practice you're very much assessing degree of inflammation evident, and again that's very much on physical examination plus or minus some blood tests” (Participant 15, consultant pediatric rheumatologist).
Theme 4: Beliefs about pain in JIA	
Subthemes	
Pain symptoms not a part of having JIA as active JIA is not painful; arthritis is not the underlying cause of pain in those who complain	“From a consultant rheumatologist point of view, they will feel quite strongly that active JIA should not be painful, and will often give that message, which kind of leads people to feel that people are not getting it, they're not believed” (Participant 4, physical therapist). “Whenever I teach other doctors about JIA I always say pain is not particularly a feature… I think a lot of patients with arthritis don't complain of much pain… often when patients complain of pain, arthritis isn't the problem” (Participant 8, consultant pediatric rheumatologist).
HCP perception that pain should be proportionate to disease activity	“When they come in, it's part of their disease process. When they're controlled, they don't have pain… it is disease activity. And then you hope that it doesn't go on to, like we've said, with chronic pain” (Participant 10, nurse). “What would make you think that a child has amplified their pain report?” (Interviewer). “Well, if their reports of pain seem out of proportion to what I find on clinical examination” (Participant 7, consultant pediatric rheumatologist).
Disregard of JIA diagnosis in light of persistent pain complaints	“And again, we just have to see chronic pains. And chronic pains are the children that have got pain… when you examine them, they've got no evidence of active arthritis” (Participant 10, nurse).
Focus on pain is unhelpful for those who have chronic pain	“With the JIAs, if I think that they may be tipping into a bit of a chronic pain and they are very focused on the pain, I'll maybe try and not talk about pain” (Participant 13, physical therapist).
Lack of evidential pain in those with seemingly inactive arthritis	“People have different opinions of chronic pain patients than of JIA. They see somebody with a real condition and JIA is an inflammatory condition, whereas chronic pain, there might not always be something to see” (Participant 17, physical therapist).

a See Table 3 for definitions.

This theme is closely related to the previous theme but shows how a reluctance to discuss pain can contribute to the low prioritization of the assessment of pain across the range of HCPs interviewed. Participants reported that they did not want to dwell on pain in clinical consultations and that pain assessment was not necessary at every rheumatology appointment.

Some participants acknowledged the importance of pain assessment but that it had a lower priority relative to other assessments of disease activity and that disease activity needs to be assessed first, even if that did not leave time for pain assessment. Functional assessments and markers of disease activity were judged to be the highest priority measurements for participants involved in managing JIA in children and young people. This again reflects participants’ attention to pain in the context of levels of inflammatory disease activity and interference with function. In some cases, participants reported that they would make assumptions about the severity of pain based solely on measures of disease activity and functional assessments. There seemed to be little awareness in these accounts that pain levels may act independently of disease activity markers.

#### Theme 4: Beliefs about pain in JIA (Table 4)

Most of the HCPs interviewed believed that persistent pain was not an intrinsic feature of JIA and that HCPs should generally avoid reinforcing the perception that it is such a feature. It was felt that active JIA was not necessarily painful, a view which may have led to patients’ accounts of pain being discredited. Several participants indicated that when patients reported pain, their JIA would not be the underlying cause. This assumption seemed to be based upon the perception that patients with arthritis do not complain about much pain. In some cases, this led to participants believing that patients with arthritis did not complain about pain while reporting that in their own practice, they rarely asked about or were reluctant to engage in discussions about pain.

Most of the accounts reflected the belief that pain symptoms caused by JIA are directly proportionate in severity to clinically observed levels of disease activity. There were also strong perceptions that if disease activity processes were controlled then the pain would consequently be reduced. Accounts and descriptions of pain that were given during the interviews tended to reflect a “medical model” of pain, that is, the view that pain stems directly from the site of disease or injury, and that pain severity is in proportion to the degree of injury. Most HCPs described managing quite a high number of children and young people with JIA whose reported pain was viewed as disproportionate in the context of the clinical examination. If children and young people reported levels of pain that were not congruent with their measures of disease activity (suggesting low levels of inflammation) then an additional or even an alternative diagnosis of chronic pain was suggested. Throughout interviews, HCPs referred to 2 distinct groups of patients in rheumatology. The first group are those with active JIA with directly proportionate pain. The second group are patients with well‐controlled JIA but for whom pain remains a problem; it was here that JIA diagnosis seemed to be decoupled from the pain experiences. In those cases, the patient's focus on pain was viewed as the underlying problem. Furthermore, there was not always evidence of the underlying disease those viewed as having chronic pain, whereas for those with JIA, the pain was referred to as more “real.”

#### Theme 5: Treatment of pain (Table 5)

**Table 5:** Themes 5 and 6, subthemes, and associated interview excerpts^a^

Themes and subthemes	Interview excerpts
Theme 5: Treatment of pain	
Subthemes	
Pharmacologic management of JIA will reduce pain levels	“If I treat the swelling, then the pain will get better, I don't really think of it as a distinct entity” (Participant 1, trainee rheumatologist). “Well I suppose in managing the disease with steroids or other management you are treating the pain” (Participant 19, physical therapist).
Over‐medicalizing the treatment of pain in JIA	“It is all about the medication. It is all about the injections. I think the hospital system is so medical” (Participant 12, occupational therapist).
Referral of patients elsewhere (therapy or specialist pain services) when pain did not respond to medication	“They (rheumatologists) are very interested in the JIAs because they can give them medicine. Some of the mechanical pain and the hyper mobility and the chronic pains they just palm them…you know, they are just not interested” (Participant 13, physical therapist). “Some consultants will spend more time asking about the pain, some will acknowledge it's there but then pass onto physiotherapy… if it's an inflammatory thing then we want to medically manage that, and if it's not then we should pass them onto physio or psychology” (Participant 21, trainee rheumatologist). Interviewer: “When you say chronic pain patient, would you describe what you mean?” Participant 6 (Nurse): “So they're patients that…every single day they're in pain with no focus… generally it's everywhere…their attendance at school or work or life activities is very low.” Interviewer: “Mm. What about if they were a JIA patient but that's under control and…?” Participant 6 (Nurse): “And…but there's still a chronic pain? I'd still do the same. Still do the same referral to the same people‐to the pain service.”
Theme 6: Undervaluing pain reports	
Subthemes	
Negative responses from HCPs towards patients with chronic pain	“With the chronic pains, I always think you can see people smiling or raising their eyebrows about it” (Participant 10, nurse). “There are times that people will roll their eyes at certain patients, because they're in pain” (Participant 18, nurse).
Undervaluing seriousness and unsympathetic responses to pain	“Arthritis pain is awful but never that awful, I don't think” (Participant 1, trainee pediatric rheumatologist).
Conscious efforts to think about the broader context of pain and consider reports seriously	“Even though in the back of your mind you are fairly certain it's gonna be just chronic pain that you're dealing with, you've also got to take it seriously” (Participant 20, physical therapist).
Difficulties trying to objectively evaluate CYP pain	“You immediately feel anxious as soon as pain comes up… it can be so hugely over‐reported and so difficult to make sense of” (Participant 9, consultant pediatric rheumatologist). “And it's really difficult…you are basically making a judgement about whether their reaction to what happened is proportionate or not” (Participant 13, physical therapist).
Questioning the credibility of pain reports, e.g., is there a financial gain, disability benefit and/or more attention from significant others	“If there's a financial reward to them having pain…they get benefits for it if they're perceived as being disabled” (Participant 12, occupational therapist). “Some patients might play on it, they get more attention” (Participant 16, occupational therapist).

a CYP = children and young people. See Table 3 for other definitions.

There was a strong belief that pharmacologic management of JIA would reduce the amount of pain that children and young people experienced. On further examination, we found that this was more evident in the accounts given by rheumatologists and less likely to appear in the therapists’ accounts. In addition, therapists were more likely to express greater concerns about an “overly‐medicalized” approach to JIA management and treatment of symptoms. This was the only theme in which we found potential evidence of discipline or profession‐specific beliefs expressed.

Study participants talked about the importance of referring JIA patients with persistent pain (which HCPs perceived as “non‐inflammatory”) to services for patients outside rheumatology. Some of the therapists perceived these referrals from rheumatologists as indicating a lack of rheumatologists’ interest in pain management. In contrast, some rheumatologists implied that while they acknowledged the presence of pain, they felt that they did not have the relevant skill set to manage noninflammatory pain. If a medical management approach of disease did not work to reduce pain, there was a view that pain could only be managed through these other, more specialized pain services.

#### Theme 6: Undervaluing pain (Table 5)

Many HCPs had created their own terms to label children and young people with persistent pain that could not be treated through medical management of arthritis, such as referring to these patients as having “chronic pains.” HCPs reported noticing negative responses from other HCPs when these patients were seen in clinic such as eye‐rolling, smiling, or raising eyebrows, behaviors that seemed to signify that these patients’ reports of pain were unconvincing. Some HCPs in the study appeared to minimize the seriousness or relevance of chronic pain and the importance of how severe suffering could be for children and young people with persistent pain.

There were suggestions that chronic pain might not always be taken seriously in clinical contexts and that it needed a conscious effort from the HCP to think about the broader context of that pain. Pain symptoms seemed to be undervalued because of the difficulties associated with evaluating pain severity objectively in children and young people. Participants perceived their role to include making judgments about participant's reactions to pain and described this challenge as anxiety provoking.

HCPs believed that there were benefits for patients or families who overreported or exaggerated the severity of pain symptoms, and this further complicated HCPs’ task of making sense of pain. These benefits could be financial reward, disability benefit, and/or more attention from significant others. These findings suggested that HCPs often questioned the credibility of patients’ pain reports and the potential advantages for each patient who reported more pain than participants thought was appropriate.

## Discussion

This is, to our knowledge, the first study to explore HCPs’ beliefs about the role of pain in JIA and the prioritization of pain assessment in patients with this long‐term condition. Findings suggest that professionals managing JIA in children and young people are largely working from a somewhat outmoded model of pain as something that should be directly and proportionately related to degree of disease activity. Alternative, more complex, and current models of pain conceptualize subjective pain experiences as the result of bidirectional interactions involving biologic, psychological, and contextual processes 27. These approaches recognize that sensory pain inputs are filtered by a variety of mechanisms, both biologic and psychological, such as genetic predispositions, prior learning, emotional status, and context. Acknowledgment of these processes was missing in the accounts of pain in children and young people with JIA given by many professionals interviewed in our study.

Our findings indicate that for some HCPs, their “personal models” of pain associated with JIA may not be congruent with research findings, which suggest that pain acts independently of levels of disease activity in JIA 7, 16, 17. A personal model of illness can be defined as an individuals’ beliefs, emotions, knowledge, experiences, and behaviors (28) and are important in shaping the conceptualizations of children and young people and their parents of the condition 30. In JIA, the importance of developing a comprehensive understanding of pain, including how and when to treat it and when to ignore or persist with activities despite pain, is essential for effective pain management.

It is apparent from our findings that understanding from pain theory and patients’ experiences have not been translated into current practice. It is important that assessment and management of pain is incorporated into clinical practice alongside the assessment and management of the disease. Our research suggests that a paradigm shift is needed in approaches to pain assessment and communication by professionals managing JIA in children and young people. Pain assessment scores can affect later pain management decisions (30). Pain may, in some instances, be best managed in other services. However, if initial presentation occurs in pediatric rheumatology then it is important that the pain management needs of these patients have been assessed and communicated before appropriate referrals are made.

Overall, our study demonstrated that HCPs’ beliefs about the role of pain in JIA may be a factor determining how or whether they feel able to support patients to manage pain symptoms. This study demonstrates that for these HCPs, pain assessment was not always a major part of clinical consultations with JIA patients. In a study by Guzman et al 12, comparison of the priorities of children and HCPs in JIA assessment found that pain was of medium importance to HCPs. However, our study finds pain and its assessment to be of low priority compared to other clinical assessments in this group of participants, and there appeared to be a reluctance for some HCPs to initiate conversations about pain. Similar to a study by Fitzcharles et al 31, our study found that HCPs concentrated on measurement of underlying disease mechanisms, as disease activity measures took precedent.

One potential limitation of the current study is that data were only drawn from participants working in the UK national health care system. It is interesting to consider our findings in the light of UK clinical practice guidelines. There are no performance standards for pain assessment in the UK 13 and no recommendation to assess pain as an indicator of disease improvement 32 or therapeutic response 33 in pediatric rheumatology. Even where a recommendation to routinely assess pain has been given as one of the standards of care for JIA 34, no guidance is given about how to assess pain.

Our research demonstrates that the recognition of pain assessment should be a higher priority in pediatric rheumatology in the UK. The findings of our study identify some of the attitudinal and practical barriers to achieving such a priority level.

## Author Contributions

All authors were involved in drafting the article or revising it critically for important intellectual content, and all authors approved the final version to be submitted for publication. Dr. Lee had full access to all of the data in the study and takes responsibility for the integrity of the data and the accuracy of the data analysis.

### Study conception and design

Lee, Rashid, Thomson, Cordingley.

### Acquisition of data

Lee, Cordingley.

### Analysis and interpretation of data

Lee, Rashid, Thomson, Cordingley.
