# Supplementary material for: “Reluctant to Assess Pain”: A Qualitative Study of Health Care Professionals’ Beliefs About the Role of Pain in Juvenile Idiopathic Arthritis
Source: Arthritis Care Res (Hoboken). 2019 Dec 27;72(1):69–77. doi: 10.1002/acr.23827 (PMC6973019; doi:10.1002/acr.23827)
Supplement: Supplementary file 1 [file ACR-72-69-s001.docx]

Supplementary Table 1: **Index of emerging themes.**

| **1** | **Skills and training:** |
| --- | --- |
| 1.1 | Lack of training in pain assessment |
| 1.2 | Recognising the need for taught pain assessment skills |
| 1.3 | HCPs developing their own skills/approaches to pain assessment |
| **2** | **Views/perceptions about significance of pain in JIA** |
| 2.1 | Hierarchy of other more ‘important’ disease variables |
| 2.2 | Pain as a feature of JIA |
| 2.3 | Chronic pain subtype vs JIA patients |
| 2.4 | Benefits/reasons for children over-reporting pain |
| 2.5 | Negative language used to describe pain in this population (e.g. not wanting to ‘dwell’ on pain) |
| **3** | **Appointment focus and pain** |
| 3.1 | Measurement of progress in other disease variables |
| 3.2 | Other disease variables as a proxy for pain measurement |
| 3.3 | Indirectly asking about/gaining information on pain |
| **4** | **Approach to pain assessment/communication** |
| 4.1 | Who approaches subject |
| 4.2 | Tools used |
| 4.3 | Importance of different facets of pain |
| 4.4 | Patient vs parental reports |
| **5** | **Pain information in subsequent treatment decisions** |
| 5.1 | Pain information doesn’t drive management |
| 5.2 | Pain information not a feature in treatment decision making |
| **6** | **Current barriers toward better assessment in JIA** |
| 6.1 | Concerns about asking about pain |
| 6.2 | Children’s fears about talking about pain |
| 6.3 | HCPs reasons for avoiding talking about pain |
| 6.4 | Need for better assessment tools |
| **7** | **Advice/education for patients and models of understanding** |
| 7.1 | Developing children’s understanding of pain mechanisms |
| 7.2 | Educating parents about pain |
| 7.3 | Pain management advice |
| 7.4 | Development of own resources |
